# Case Report: Effective management of adalimumab-induced acquired hemophilia A with the CyDRI protocol

**DOI:** 10.3389/pore.2024.1611720

**Published:** 2024-05-23

**Authors:** Andrea Ceglédi, Árpád Bátai, János Dolgos, Mónika Fekete, László Gopcsa, Viktória Király, Gergely Lakatos, György Nagy, Zsuzsanna Szemlaky, Andrea Várkonyi, Beáta Vilimi, Gábor Mikala, Imre Bodó

**Affiliations:** ^1^ Departments of Hematology and Stem Cell Transplantation, South Pest Central Hospital, National Institute of Hematology and Infectious Diseases, Budapest, Hungary; ^2^ Department of Public Health, Semmelweis University, Budapest, Hungary; ^3^ Doctoral College, Health Sciences Program, Semmelweis University, Budapest, Hungary; ^4^ Szent György Fejér County University Hospital, Székesfehérvár, Hungary; ^5^ Department of Rheumatology and Clinical Immunology, Department of Internal Medicine and Oncology, Semmelweis University, Budapest, Hungary; ^6^ Heart and Vascular Center, Semmelweis University, Budapest, Hungary; ^7^ Hospital of the Hospitaller Order of Saint John of God, Budapest, Hungary; ^8^ School of PhD Studies, Semmelweis University, Budapest, Hungary; ^9^ Department of Internal Medicine and Hematology, Semmelweis University, Budapest, Hungary; ^10^ Department of Hematology and Medical Oncology, Emory University, Atlanta, GA, United States

**Keywords:** adalimumab, acquired hemophilia A, bleeding disorder, immunosuppression, rheumatoid arthritis

## Abstract

**Introduction:**

Acquired Hemophilia A (AHA) is a rare autoimmune disorder characterized by the emergence of inhibitors that specifically target coagulation Factor VIII, frequently resulting in severe bleeding episodes.

**Methods:**

We conducted a retrospective analysis of the medical records of a 68-year-old male patient who presented with adalimumab-induced AHA.

**Results:**

The patient received adalimumab, a tumor necrosis factor inhibitor antibody, as part of his treatment for rheumatoid arthritis. The patient’s clinical journey, characterized by intense bleeding and coagulopathy, was effectively managed with the application of recombinant Factor VIIa (rFVIIa) and the CyDRi protocol.

**Discussion:**

The case emphasizes the importance of prompt coagulation assessment in patients with bleeding symptoms receiving disease-modifying therapy for rheumatoid arthritis that includes adalimumab therapy, considering the rare yet life-threatening nature of AHA. Additionally, this report provides an extensive review of the existing literature on drug-induced AHA, with a special emphasis on cases linked to immunomodulatory medications. Through this two-pronged approach, our report aims to enhance understanding and awareness of this severe complication among healthcare providers, promoting timely diagnosis and intervention.

## Introduction

Acquired hemophilia A (AHA) is an autoimmune disorder characterized by the spontaneous emergence of autoantibodies against coagulation Factor VIII, leading to a rare, yet serious bleeding disorder [1]. AHA, while rare, poses a significant clinical challenge due to its severe bleeding manifestations and potentially life-threatening complications [1]. The incidence of AHA is estimated to be approximately 1–1.78 cases per million individuals per year [1–4]. The condition may arise at any age, though incidence is notably higher in older populations [1, 3, 5]. This age-related propensity could be attributed to various factors, including age-associated changes in the immune system.

While the root cause of AHA is frequently idiopathic or associated with autoimmune diseases or malignancies, an increasing trend is observable with more cases attributable to certain medications, especially those with immunomodulatory properties [1, 3]. This paper aims to delve deeper into this association by presenting a detailed case report of an adalimumab-induced AHA and conducting a comprehensive review of the literature, thereby illuminating this uncommon clinical challenge and discussing its management strategies.

Adalimumab is a fully human monoclonal antibody that targets and neutralizes tumor necrosis factor-alpha (TNF-α), a key cytokine involved in the inflammatory process. As an immunomodulatory agent, adalimumab is extensively used in the treatment of various autoimmune diseases, such as rheumatoid arthritis, psoriatic arthritis, ankylosing spondylitis, Crohn’s disease, ulcerative colitis, and psoriasis. Its mode of action involves dampening the inflammatory response, which is beneficial in controlling the symptoms of these autoimmune conditions. However, by modifying the immune response, adalimumab can potentially induce a range of immune-mediated adverse effects, including the rare development of AHA [6–8].

In this context, the presented case report of adalimumab-induced AHA is particularly pertinent, offering valuable insights into the complex interplay between immunomodulatory therapy and autoimmune responses leading to coagulation disorders. By reviewing related literature, this paper aims to enhance understanding of AHA’s pathogenesis, clinical presentation, diagnostic challenges, and therapeutic approaches, specifically focusing on its association with adalimumab.

## Methods

We conducted a retrospective analysis of the medical records of a 68-year-old Caucasian male who presented with severe AHA-related bleeding and subsequent immunosuppressive therapy. The publication of this report was approved by the ethics committee of the South Pest Central Hospital/National Institute of Hematology and Infectious Diseases. The patient provided written informed consent for the analysis of his medical records, which included comprehensive data such as laboratory results, imaging findings, surgical reports, and detailed medication records.

Data were collected from the patient’s medical records, including laboratory test results, imaging studies, and medication administration records. We closely followed the patient’s clinical state from his initial admission through to the current date, carefully evaluating his response to treatment based on the evolution of his bleeding symptoms, laboratory findings, and any adverse reactions observed. The time course of changes in coagulation parameters, including FVIII activity, was depicted graphically. This retrospective study, however, is not without limitations. There is the inherent possibility of missing or incomplete data in such analyses, and establishing a direct causal link between the patient’s clinical trajectory and the administered treatments is challenging. Additionally, as this report centers on a single patient’s experience, its findings may not be broadly applicable to other individuals with similar medical conditions.

## Results

### Patient background and initial presentation

The patient was urgently admitted to our department on 23 December 2022. He presented with circulatory collapse caused by massive gastrointestinal bleeding manifested as melena, severe anemia, macroscopic hematuria, and extensive ecchymoses and hematomas across his body.

The patient’s medical history included long-standing controlled hypertension (diagnosed in 1996), seropositive rheumatoid arthritis (diagnosed in April 2013), a renal stone (2018), sigma diverticulosis confirmed by colonoscopy, and pulmonary fibrosis (diagnosed in 2021). Additionally, he had a history of lumbar spine surgery (discectomy) and cervical lymph node biopsy (in 2021), none of which were complicated by bleeding. The patient had no personal or family history of bleeding disorders.

Of particular note is the history of his rheumatoid arthritis management. Since 2013, he has been receiving intermittent NSAID therapy in addition to a series of immunomodulatory drugs. His disease-modifying treatment journey included methotrexate, followed by leflunomide, participation in a lupin therapy study in 2017, and sulfasalazine in 2019. The inefficacy of prior therapies ultimately culminated in the initiation of adalimumab treatment in April 2022, administered as 40 mg subcutaneously every 2 weeks. The timing and nature of his presentation raised concerns about the potential role of adalimumab in his current clinical state, especially given the absence of a personal or family history of bleeding disorders.

### Diagnostic workup and findings

In the weeks preceding admission, the patient experienced a syncopal episode due to gastrointestinal bleeding. Subsequent evaluations at another institution, including gastroscopy and colonoscopy, failed to identify the bleeding source, though gastritis, diverticulosis, and polyps were noted. Capsule endoscopy revealed ulcers and angiodysplasias in the jejunum, which were treated with argon plasma coagulation and hemoclips. Despite repeated transfusions, PPI inhibition, and other interventions, the patient’s anemia persisted, and the bleeding did not stop.

His coagulation parameters were: INR: 0.97, PT: 8.6 s (normal range: 9.4–12.5 s), APTT: 105.8 s (normal range: 28–40 s), mixing test: APTT: 50 s, APTT 2-h incubation: 110.3 s, TT: 15.7 s (normal range: 10.3–16.6 s), Fibrinogen: 6.18 g/L (normal range: 2.76–4.71 g/L), FVIII activity: under 0.5% (normal range: 50%–150%), FVIII inhibitor level: 29.4 BU/mL. Tests for lupus anticoagulant and anticardiolipin antibodies were negative. Acquired hemophilia A was diagnosed.

### Treatment and management

**FIGURE 1:**
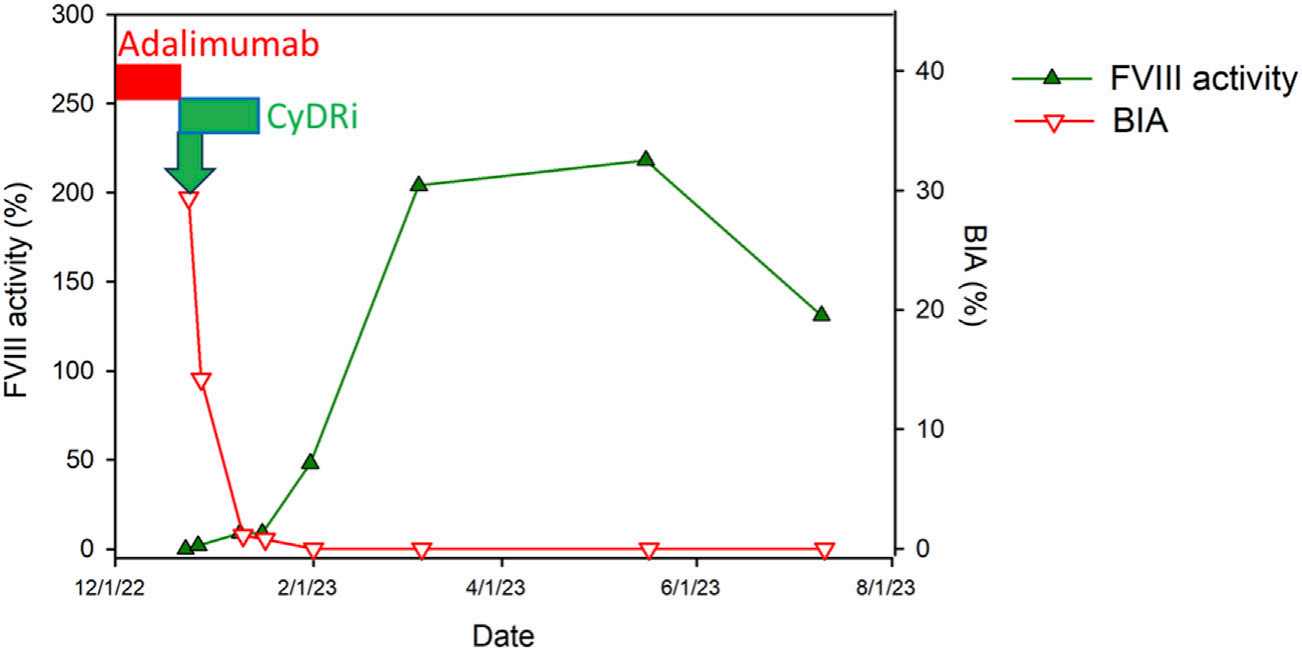
Time course of Factor VIII dynamics following CyDRi treatment in a male patient with adalimumab-induced AHA. This figure displays the temporal correlation between the administration of the CyDRi immunosuppressive regimen (comprising cyclophosphamide, dexamethasone, and rituximab) and subsequent alterations in Factor VIII (FVIII) inhibitor levels (represented by the Bethesda titer in red) as well as FVIII activity (indicated in green). These changes are mapped over time to demonstrate the patient’s hematological response to the treatment. Key treatment intervals are marked by bars: the period of adalimumab administration is highlighted in red, while the initiation of the CyDRi regimen, after the discontinuation of adalimumab, is denoted in green. This visual representation aids in understanding the efficacy of the CyDRi regimen in altering FVIII parameters post-adalimumab therapy.

For immediate bleeding control, the patient received an initial dose of rFVIIa (90 µg/kg every 4 h), which was gradually adjusted, leading to the complete cessation of bleeding 13 days post-admission. Concurrently, to eradicate the inhibitor, he was placed on the CyDRi protocol comprising cyclophosphamide (1,000 mg on days 1 and 22), dexamethasone (40 mg on days 1, 8; 20 mg on days 15, and 22), and rituximab (100 mg on days 1, 8, 15, and 22) (Figure 1).

Moreover, he developed a febrile condition, later identified as an ESBL *Klebsiella pneumoniae* infection, necessitating a tailored antibiotic regimen (ertapenem-amikacin, meropenem) and modifications to the CyDRi protocol (dexamethasone dose was reduced to 20 mg on days 15 and 22). Additional interventions included emergency IVIG treatment (2 × 60 g i.v.) and a single administration of G-CSF (filgrastim 48 ME s.c. inj./day) due to febrile neutropenia. Notably, adalimumab was discontinued and marked as contraindicated for future use.

### Outcome and follow-up

The patient’s condition gradually stabilized, allowing for a discharge 23 days post-admission. Remarkably, coagulation parameters completely normalized at 39 days following admission and have remained stable since.

## Discussion

Between 3% and 5% of AHA cases are linked to drug exposure, where certain medications act as a trigger for the immune system to produce antibodies against Factor VIII [9]. While the exact mechanism remains unclear, it is hypothesized that these drugs may alter the immune response, trigger the production of antibodies that cross-react with Factor VIII, or alter the structure of Factor VIII, making it appear foreign to the immune system. A comprehensive global pharmaco-epidemiologic study has identified 14 drugs linked to the onset of AHA [10]. This catalog encompasses medications, such as the antiplatelet drug clopidogrel [11, 12], along with alemtuzumab [13–18] and omalizumab [19], monoclonal antibodies used in treating specific leukemia types and allergic asthma, respectively [10]. Other drugs that have been associated with AHA include penicillins [20–22], interferons [23–25], chlorpromazine [26], acetaminophen and chlorpheniramine [27] and anti-cancer drugs including fludarabine and imatinib [10].

Immunomodulatory agents used to treat autoimmune diseases and malignancies are particularly interesting with respect to the genesis of drug-induced AHA. These drugs, through their action on the immune system, may unintentionally stimulate the production of inhibitors against Factor VIII. The medical literature has documented instances where the onset of AHA has been linked to the use of alemtuzumab (anti-CD52) [13–18, 28–30], nivolumab (PD-1 inhibitor) [31–33] and TNFα inhibitors, particularly etanercept [34] and infliximab, which are commonly prescribed for various autoimmune disorders. These drugs suppress the inflammatory response mediated by TNFα, which may inadvertently lead to the emergence of AHA. This array of medications, while instrumental in treating autoimmune diseases, underscores a critical challenge due to their potential side effect of inducing AHA. It emphasizes the need for healthcare professionals to be vigilant for signs of bleeding disorders in patients undergoing these treatments, balancing the therapeutic benefits with the risks of adverse hematological events.

A striking aspect of drug-induced AHA is its demographic and temporal patterns. The median age of patients experiencing AHA onset due to drug exposure is typically around the age of 75 years [10]. The increased prevalence of autoimmunity in older individuals may be attributed to several factors related to the aging process, including immunosenescence [35], chronic low-grade sterile inflammation (“inflammaging” [36, 37]), cumulative environmental exposure [38], clonal hematopoiesis of indeterminate potential (CHIP) [39], age-related thymus involution and hormonal changes. Importantly, there is generally a median period of about 30 days from the start of the implicated medication to the emergence of AHA symptoms [10]. Alarmingly, about 10% of these cases have led to fatal outcomes [10]. This underscores the complexity of drug-induced AHA, highlighting the need for vigilant monitoring of patients, especially the elderly, who start new medications with known associations to AHA.

In recent years, efforts have been made to elucidate the immunogenetic background predisposing individuals to AHA. Studies have identified genetic variations within genes of the HLA cluster, KLRK1, and CTLA4, revealing notable associations with AHA [40–43]. These findings represent a crucial advancement, shedding light on specific alleles potentially implicated in AHA susceptibility for the first time. Further molecular and functional investigations are warranted to elucidate their precise contributions. Ultimately, such insights hold promise for enhancing AHA diagnosis and prognosis, emphasizing the significance of genetic factors in its pathogenesis.

Drug-induced AHA is a serious clinical concern due to the acute and severe nature of the bleeding episodes it can cause. These bleeding events can occur in various parts of the body, including muscles, skin, and internal organs, and can be life-threatening if not promptly diagnosed and appropriately treated. The two-pronged management of AHA involves controlling the bleeding episodes and eradicating the inhibitors through various therapeutic strategies.

### Adalimumab and AHA

Adalimumab, classified as a disease-modifying antirheumatic drug, is a fully human, high-affinity, recombinant monoclonal antibody, which functions by neutralizing TNFα, a key molecule involved in inflammatory processes. While effective in managing various autoimmune disorders, its use is associated with a range of potential side effects. These include a heightened risk of serious infections, increased likelihood of certain cancers, and the possibility of anaphylactic reactions [44, 45]. Additionally, there is a concern for the reactivation of hepatitis B in carriers [46, 47]. The drug may also trigger new onset or worsen existing demyelinating diseases, such as multiple sclerosis [48–53], and has been linked to heart and liver failure [54, 55], as well as rare cases of autoimmune hemolytic anemia [56–58].

Our case adds to the emerging evidence that the immunomodulatory effect of adalimumab can result in the production of Factor VIII inhibitors, leading to AHA. An important aspect of adalimumab-induced AHA is its rarity. To date, only three other documented cases exist in medical literature [6, 8]. This scarcity underscores the challenge in diagnosing and managing adalimumab-induced AHA. Each of these cases provides valuable insights into the clinical presentation, diagnosis, and treatment outcomes of AHA in patients under adalimumab therapy. Lieberman and Burkholder described a patient who presented with AHA 3 years after beginning treatment with adalimumab for necrotizing scleritis [7]. Arthanari et al reported a fatal case of adalimumab-induced AHA in a patient with rheumatoid arthritis [8]. Yamaguchi et al. described adalimumab-induced AHA and fulminant diabetes mellitus in a patient with psoriatic arthritis [6]. The limited number of reported cases involving adalimumab-induced AHA exhibit several shared characteristics, most notably the abrupt emergence of bleeding symptoms. Given the rarity of these occurrences, each reported instance becomes crucially important for deepening our understanding of this pathology. The collection and comparative analysis of more cases are vital in identifying and confirming common patterns and features associated with adalimumab-induced AHA. This could include typical patient demographics most at risk, underlying health conditions, and the effectiveness of various treatment strategies.

Given the serious nature of AHA and the challenges associated with its diagnosis, clinicians prescribing adalimumab should be aware of its potential to induce AHA. Early detection and intervention are crucial for patient outcomes. This necessitates a high degree of suspicion, especially in patients presenting with unexplained bleeding symptoms, and a thorough understanding of the patient’s medical history and current medication regimen.

### Clinical management

The diagnosis and management of drug-induced AHA typically follow the same protocols as those used for idiopathic cases [59–62], largely because establishing a definitive causal link to a specific drug often remains a matter of strong clinical suspicion rather than absolute confirmation. The key exception in this approach is the immediate cessation of the implicated drug and advising against its future use. Laboratory tests crucial for diagnosis include measuring activated partial thromboplastin time (APTT), Factor VIII (FVIII) activity, and conducting Bethesda assay or anti-FVIII ELISA. Additionally, for more nuanced therapeutic decision-making, the diagnostic process may be enhanced by using the modified Bethesda assay to measure anti-porcine inhibitor levels.

Treatment of drug-induced AHA is primarily centered around controlling acute bleeding episodes and eliminating the inhibitors against Factor VIII. This necessitates a strategic combination of hemostatic agents and immunosuppressive therapy. The approach to hemostatic treatment typically includes the use of recombinant Factor VIIa (rFVIIa), activated prothrombin complex concentrate (APCC), and recombinant porcine Factor VIII (rpFVIII). In certain specific clinical scenarios, the use of plasma-derived human FVIII concentrates, desmopressin, or a combination of plasmapheresis with immunoabsorption may be considered. Moreover, the potential role of emicizumab in treating acquired hemophilia A is currently being evaluated in ongoing clinical trials.

Parallel to hemostatic management, the second crucial aspect of treatment aims at the eradication of inhibitors through immunosuppressive therapy. The most commonly used medications for this purpose include corticosteroids, cyclophosphamide, and rituximab, with other agents like cyclosporine, vincristine, azathioprine, and mycophenolate mofetil being used less frequently but still relevant in certain cases. However, the choice of treatment in drug-induced AHA must be meticulously considered, particularly in patients with underlying conditions for which the implicated drug was initially prescribed. The immediate suspension of the suspected drug and advising against its future use are critical steps.

#### Innovative combined immunosuppressive treatment for AHA: the efficacy and low toxicity of the CyDRi regimen

In our recent study, we have made a notable advancement in treating AHA through the innovative CyDRi regimen [63]. This approach combines the use of cyclophosphamide, dexamethasone, and low-dose rituximab in pulse doses [63]. It stands out for its rapid effectiveness and minimal toxicity, particularly in older patients with AHA. The specifics of the CyDRi regimen include administering 1,000 mg of cyclophosphamide on days 1 and 22, 40 mg of dexamethasone on days 1, 8, 15, and 22, and 100 mg of rituximab on the same schedule [63]. The CyDRi treatment distinguishes itself through several key strategies: employing dexamethasone as the steroid component, concurrently using all three drugs from the outset, the specific timing of dosing for each drug, and its application in both resistant and recurrent cases of the disease. This regimen has demonstrated remarkable effectiveness across various types of AHA, achieving a 96.8% rate of complete remission and an overall survival rate of 90.6% [63]. These results significantly surpass those typically seen with traditional sequential treatment approaches. Accordingly, Collins et al. conducted a comparative analysis of various immunosuppressive protocols using data from 331 patients enrolled in the prospective EACH2 registry [64]. Their findings revealed that combining steroids with cyclophosphamide alone led to a significantly lower rate of stable complete remission (70%), whereas either steroids alone (48%) or rituximab-based regimens (59%) yielded even worse outcomes [64]. The median time to achieve complete remission was approximately 5 weeks for protocols involving steroids, regardless of the inclusion of cyclophosphamide, while rituximab-based regimens required nearly twice as long [64]. The time to reach complete remission using the CyDRi regimen was reported to be in a similar range [63]. Furthermore, the favorable toxicity profile of the CyDRi regimen significantly contributes to its superior outcomes, rendering it a highly effective and less harmful option for immunosuppressive therapy, particularly in older AHA patients. In our retrospective analysis of data from 32 AHA patients treated with the CyDRi regimen, only one patient developed *Clostridium difficile* colitis during hospitalization, and another patient experienced *Klebsiella* cystitis, which responded well to antibiotic treatment. Taken together, the case presented further reinforces our earlier findings, demonstrating the effectiveness of the CyDRi regimen in treating drug-induced AHA cases, such as those associated with adalimumab therapy. These results suggest that the CyDRi regimen could potentially reshape future treatment protocols for various types of AHA.

## Conclusion

The anti-TNFα drug adalimumab stands as a pivotal development in the management of autoimmune diseases, offering substantial benefits to patients suffering from these conditions. However, its association with AHA, albeit infrequent, demands careful attention and vigilance.

The presented case illustrates the complexity of diagnosing and managing drug-induced AHA, underscoring the importance of considering this rare but serious complication in patients presenting with unexplained bleeding, especially when under immunomodulatory therapy such as adalimumab. This paper underscores the critical need for prompt and thorough evaluation of coagulation parameters in patients undergoing adalimumab treatment who experience unexplained bleeding episodes. This necessity holds true even when the bleeding source appears to be locally identifiable. Early and accurate diagnosis, followed by immediate and appropriate treatment, is imperative in cases of severe bleeding induced by acquired inhibitors against Factor VIII. Such proactive measures are vital not only to manage the immediate risks associated with AHA but also to mitigate the long-term complications of this rare yet potentially life-threatening condition. Given the wide use of adalimumab in treating various autoimmune disorders, it is vital to draw the attention of a diverse range of specialists—including immunologists, rheumatologists, and gastroenterologists—to this rare but serious yet treatable complication. Awareness among these specialists is essential to ensure timely diagnosis and treatment, thereby minimizing the risk of significant morbidity and mortality associated with AHA. Recognizing and addressing this complication early can greatly enhance patient safety, ensuring the therapeutic benefits of adalimumab in managing autoimmune conditions are not overshadowed by the risk of adverse hematological events.

## Data Availability

The original contributions presented in the study are included in the article/supplementary material, further inquiries can be directed to the corresponding author.
